# Direct Detection Electron Energy-Loss Spectroscopy: A Method to Push the Limits of Resolution and Sensitivity

**DOI:** 10.1038/s41598-017-07709-4

**Published:** 2017-08-15

**Authors:** James L. Hart, Andrew C. Lang, Asher C. Leff, Paolo Longo, Colin Trevor, Ray D. Twesten, Mitra L. Taheri

**Affiliations:** 1 Department of Materials Science and Engineering, Drexel University, Philadelphia, Pennsylvania 19104 USA; 2 Analytical Projects R&D Gatan, Pleasanton, California 94566 USA

## Abstract

In many cases, electron counting with direct detection sensors offers improved resolution, lower noise, and higher pixel density compared to conventional, indirect detection sensors for electron microscopy applications. Direct detection technology has previously been utilized, with great success, for imaging and diffraction, but potential advantages for spectroscopy remain unexplored. Here we compare the performance of a direct detection sensor operated in counting mode and an indirect detection sensor (scintillator/fiber-optic/CCD) for electron energy-loss spectroscopy. Clear improvements in measured detective quantum efficiency and combined energy resolution/energy field-of-view are offered by counting mode direct detection, showing promise for efficient spectrum imaging, low-dose mapping of beam-sensitive specimens, trace element analysis, and time-resolved spectroscopy. Despite the limited counting rate imposed by the readout electronics, we show that both core-loss and low-loss spectral acquisition are practical. These developments will benefit biologists, chemists, physicists, and materials scientists alike.

## Introduction

Electron energy-loss spectroscopy (EELS) in the transmission electron microscope (TEM) is an extremely powerful characterization tool, offering spatially resolved data on elemental composition, chemical bonds, optical properties, and vibrational modes. This technique has seen great advancement in the last decade primarily due to transformative instrumentation development. Monochromated electron sources^1–3^ and high resolution spectrometers^4, 5^ improve energy resolution more than an order of magnitude, bettering energy-loss near edge structure (ELNES) analysis^6^ and enabling the study of phonons^3, 7^ and optical properties^8^. Spherical aberration corrected electron probes greatly enhance spatial resolution and probe current density^9^, allowing almost routine atomic-scale chemical and oxidation state mapping^10, 11^. The electron sensors used for EELS have also evolved, with large improvements in speed, resolution, and noise achieved since CCD-based parallel-EELS became the industry standard^12, 13^. However, these advancements of the electron sensor have occurred incrementally, and detector design has not significantly changed in several decades. Separate from the field of electron spectroscopy, radiation-hard direct detection (DD) sensors, diverging from the conventional detector design, were developed for the transmission electron microscope (TEM)^14, 15^. DD offers substantial improvements in resolution and signal-to-noise ratio (SNR)^16–18^. These sensors have shown great potential for *in situ* microscopy^19, 20^ and have revolutionized low-dose analysis, lifting single-particle cryo-TEM to the Nature Methods 2015 Method of the Year^21–25^. Application of this new generation of DD sensors to electron spectroscopy^26, 27^ may yield similarly exciting results.

**Figure 1:**
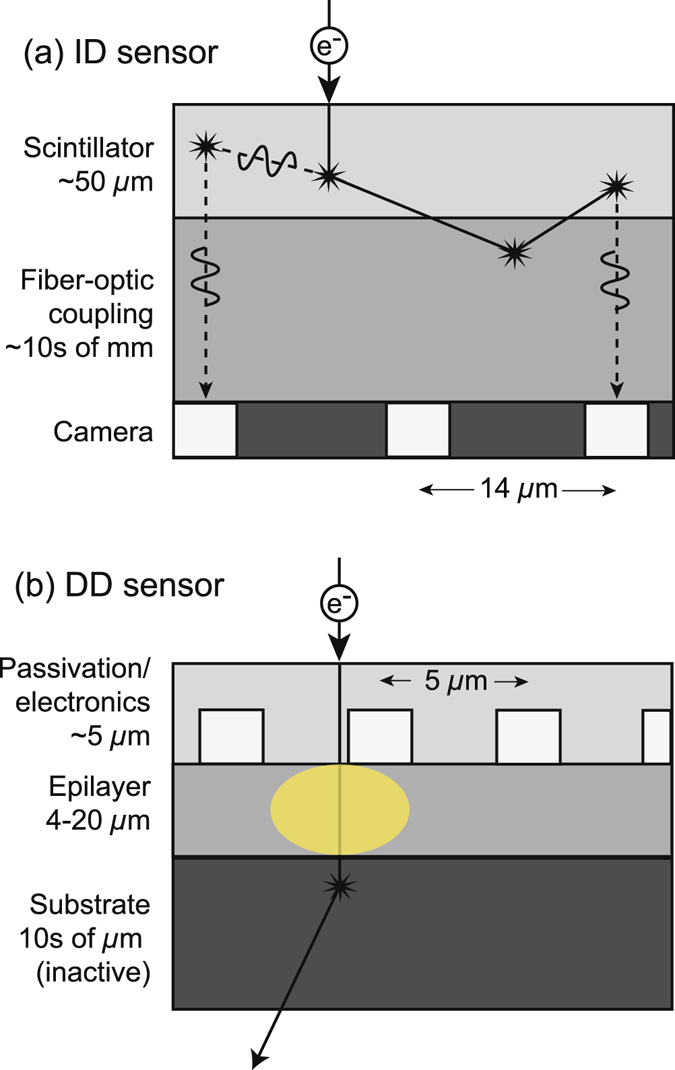
Qualitative comparison of (**a**) ID sensor and (**b**) back-thinned, front-illuminated MAPS DD sensor. Each schematic shows a single primary electron passing through the sensor. Solid lines represent electron trajectories. Dotted lines represent photon trajectories. Stars represent scattering events. The shaded yellow region represents electron-hole pair generation. The pixel pitches correspond to the Gatan US1000FTXP and Gatan K2 for the ID and DD sensor, respectively. Layer thicknesses for the DD sensor are taken from ref. 37, and do not necessarily correspond to the K2.

The considerable performance advantage offered by DD technology – and potential benefits for EELS – is understood through comparing sensor design. Conventional detectors used for EELS, here termed indirect detection (ID) sensors, consist of three layers: a scintillator to convert electrons to photons, an optical (lens or fiber) coupling, and a digital camera (typically CCD or CMOS) [Fig. 1(a)]. Electron and photon scattering within the scintillator (and electron backscattering from the fiber-optic) causes signal delocalization, increasing the detector point spread function (PSF) and reducing resolution^28^. To mitigate the effect of the sensor PSF on high-resolution EELS, higher energy magnifications must be used, which in turn reduce the energy field-of-view (FOV). This trade off often prevents simultaneous elemental mapping, which requires a large energy FOV, and chemical bond analysis, requiring high energy resolution. Additionally, acquisition with an ID sensor introduces multiple noise sources, primarily arising from electronic read-out of the CCD and the statistics of photon generation/collection^29^. This added noise necessitates extended exposures to obtain spectra of sufficient SNR. Longer exposures can prohibit the study of highly beam sensitive samples^30, 31^ and potentially influence results from nominally stable materials^32, 33^. For atomic resolution characterization where even small amounts of sample drift are problematic, extended exposures are not feasible, and the added noise can prevent quantitative data analysis^34^. For *in situ* EELS, added noise from ID sensors worsen time resolution, preventing dynamic spectroscopic tracking of certain chemical and physical processes.

There is a clear need for a low noise, high resolution sensor for EELS, and the current generation of commercial DD sensors – monolithic active pixel sensors (MAPS)^35^ – seem poised to fill this void. The MAPS design consists of a passivation/electronics layer, epilayer, and substrate. When incident electrons pass through the lightly-doped epilayer, electron-hole pairs are generated and collected to form the signal [Fig. 1(b)]. Back-thinning of the substrate allows electrons to be transmitted with minimal backscatter probability, greatly decreasing signal delocalization and significantly improving detector resolution^36, 37^. With DD, the detected signal per incident electron is large compared to inherent detector noise. This favorable SNR ratio, coupled with high speed CMOS electronics, enables counting of individual primary electrons. Electron counting essentially eliminates electronic read-out noise and the noise arising from variations in the energy deposited per incident electron; electron counting dramatically improves sensor performance^36, 38^.

In this article, we evaluate the potential of electron counting with DD for EELS by directly comparing the Gatan K2 Summit^38, 39^ (direct detection MAPS in counting mode) and the Gatan US1000FTXP (conventional scintillator/fiber-optic/CCD design). The two sensors are mounted within the same Quantum Gatan Imaging Filter^12^ (see Methods), allowing comparison under equivalent operating conditions. We find that the DD sensor out performs the ID sensor, providing improvements in energy resolution, energy FOV, and spectral detective quantum efficiency (DQE). We show that these benefits greatly improve EELS spectrum image (SI) acquisition, and we discuss implications for low-dose chemical mapping, *in situ* EELS, and trace element analysis. Concerns associated with electron counting for EELS, namely limits on electron arrival rate, are also considered.

## Results and Discussion

### Energy Resolution and Energy Field of View

EELS energy resolution at the detector is critical for the analysis of oxidation state and bonding environments, and as we demonstrate below, energy resolution may be improved with DD. To understand measured energy resolution, we begin with the inherent energy-loss function *E*_*LF*_ of a given sample. This continuous function is first convolved by the energy spread *E*_*S*_ of the electron source and then, upon striking the detector, is binned (pixelated or discretized) into a discrete signal according to the detector dispersion in eV/channel. Lastly, this signal is convolved by the detector line spread function *LSF*

1 $$R={E}_{LF}\otimes ({E}_{S}\otimes LSF)={E}_{LF}\otimes ZLP$$

where *R* is the recorded spectrum and ⊗ is the convolution symbol. In equation (1), it is apparent that the sample’s inherent *E*_*LF*_ is smoothed by both the electron source energy width and the response of the detector, though the extent that the detector *LSF* affects resolution is strongly dependent on the working dispersion. For sufficiently high dispersion (high by conventional nomenclature but small in units of eV/channel), the electron source energy spread is much broader than the extent of signal spreading between energy channels, and detector *LSF* convolution has a negligible effect on the recorded signal. In this limit, the EELS system reaches its ultimate energy resolution, which is sensor independent and determined by *E*_*S*_. When the dispersion is reduced and the energy per detector channel is comparable to *E*_*S*_, signal spreading between detector channels will influence, or entirely limit, the measured energy resolution. Since lower dispersions improve energy FOV and the SNR of spectra (due to the same number of electrons being collected over fewer channels thus increasing counts per channel), it is often advantageous to operate within this detector-limited regime.

When there is no energy loss, the *E*_*LF*_ in equation (1) becomes a delta function and the term in parenthesis becomes the experimentally measured zero loss peak (ZLP) recorded at the given detector conditions. This experimentally measured, dispersion-dependent kernel provides a reasonable measure for EELS energy resolution, but due to the change of the angular distribution of the electrons entering the spectrometer at nonzero energy losses, the relationship is only approximate. Still, the ZLP full width at half maximum (FWHM) is a common measure of system energy resolution.

**Figure 2:**
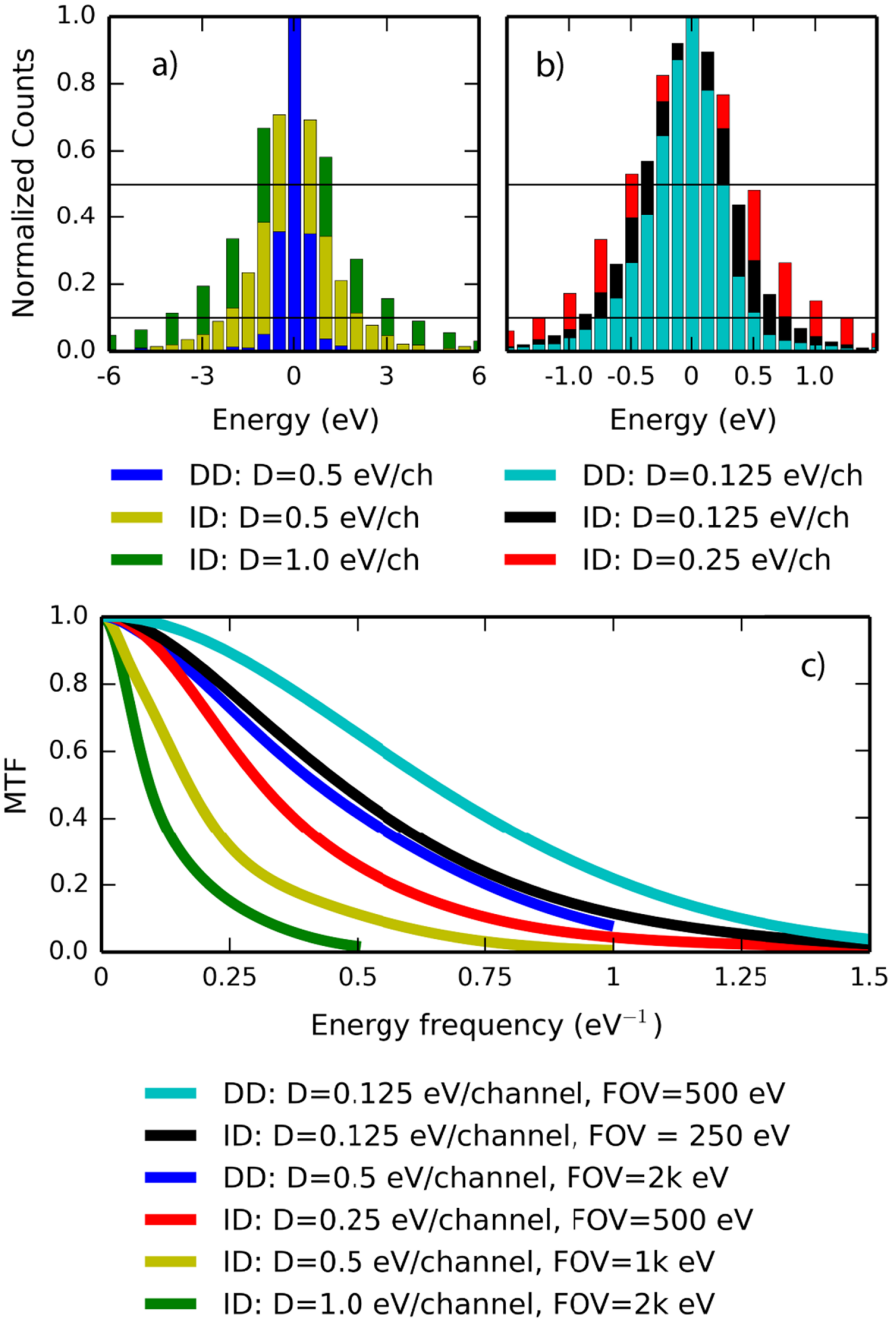
Comparison of measured EELS energy resolution with both sensors. The ZLP and MTF for the DD sensor are shown together with the ID sensor operated at equivalent dispersion and energy field of view.

Figure 2(a) and (b) show recorded ZLPs from the DD sensor operated at dispersions of 0.5 and 0.125 eV/channel, respectively. For comparison to each ZLP recorded with the DD sensor, data acquired with the ID sensor operated at an equivalent dispersion and an equivalent energy FOV is overlaid. The electron source energy spread was kept constant at 0.6 eV (measured with the DD sensor at a dispersion of 0.05 eV/channel). Table 1 shows extracted FWHM and full width at tenth maximum (FWTM) values. For all ZLPs acquired with the ID sensor, the FWHM is larger than the source energy spread, indicating a decrease in energy resolution caused by the detector. Conversely, the DD sensor records ZLP FWHMs which are less than the energy resolution of the electron source. While the narrow ZLP FWHMs recorded with the DD sensor indicate an improvement in performance, we note that the system resolution can not be less than the energy spread of the electron source, nor can the energy resolution be less than 2–3× the working dispersion owing to sampling rate considerations. The ZLP FWHMs recorded with the DD sensor that are less than the system resolution are a result of pixilation and aliasing which act to sharpen the discretely recorded spectrum.

**Table 1:** FWHM and FWTM values extracted from the data shown in Fig. 2.

Sensor	Fig. 2(a)			Fig. 2(b)		
	DD	ID	ID	DD	ID	ID
Dispersion (eV/channel)	0.5	1.0	0.5	0.125	0.25	0.125
Energy FOV (eV)	2 k	2 k	1 k	500	500	250
FWHM (eV)	0.5	3.0	1.5	0.5	1.0	0.75
FWTM (eV)	1.5	8.0	4.5	1.375	2.5	1.75

Comparing sensor performance at a dispersion of 0.5 eV/channel, the ZLP FWHM for the DD sensor is only 1/3 that of the ID sensor, demonstrating a large improvement in performance. At a dispersion of 0.125 eV/channel, the difference between sensors is reduced, though DD still offers a moderate improvement in measured energy resolution. We also compare energy resolution between sensors at constant energy FOV, which is determined by the detector pixel count multiplied by the dispersion. The K2 has 3710 pixels along the axis of energy dispersion while the US1000FTXP has 2048, thus the ID sensor must be operated at approximately D_ID_ = 2 × D_DD_ to obtain the same FOV. We note that this 1:2 ratio is specific to the experimental set up; there are 10k^2^ ID sensors^40^ as well as sub-4k^2^ DD sensors. The salient point is that the K2 pixel size is greatly reduced compared to the US1000FTXP (5 μm pitch compared to 14 μm pitch) which grants increased energy FOV given a fixed detector area. As evident in equation (1), changing the ID sensor’s dispersion to match the DD sensor’s FOV will necessarily reduce the measured energy resolution. For the comparison between DD and ID at a FOV of 500 eV, the ZLP FWHM of the DD sensor is 1/2 that of the ID sensor. For the comparison at a FOV of 2k eV, the DD sensor’s ZLP FWHM is 1/6 that of the ID sensor. When operated at equivalent FOV, DD provides greatly enhanced energy resolution.

Taking the Fourier transform of the ZLP provides an approximation of the modulation transfer function (MTF), a more robust measure of energy resolution than the ZLP FWHM. The MTF represents the system’s (including source and sensor contributions) amplitude response to sinusoidal signals of varying energy frequency *f* in units of eV^−1^; an ideal system has an MTF of unity. The MTFs are shown in Fig. 2(c) as a function of energy frequency. We highlight the finding that the MTF for the DD sensor at 0.5 eV/channel is superior to the ID sensor at 0.25 eV/channel and comparable to the ID sensor at 0.125 eV/channel. For this similar energy resolution, the DD sensor possesses 8 × the energy FOV.

**Figure 3:**
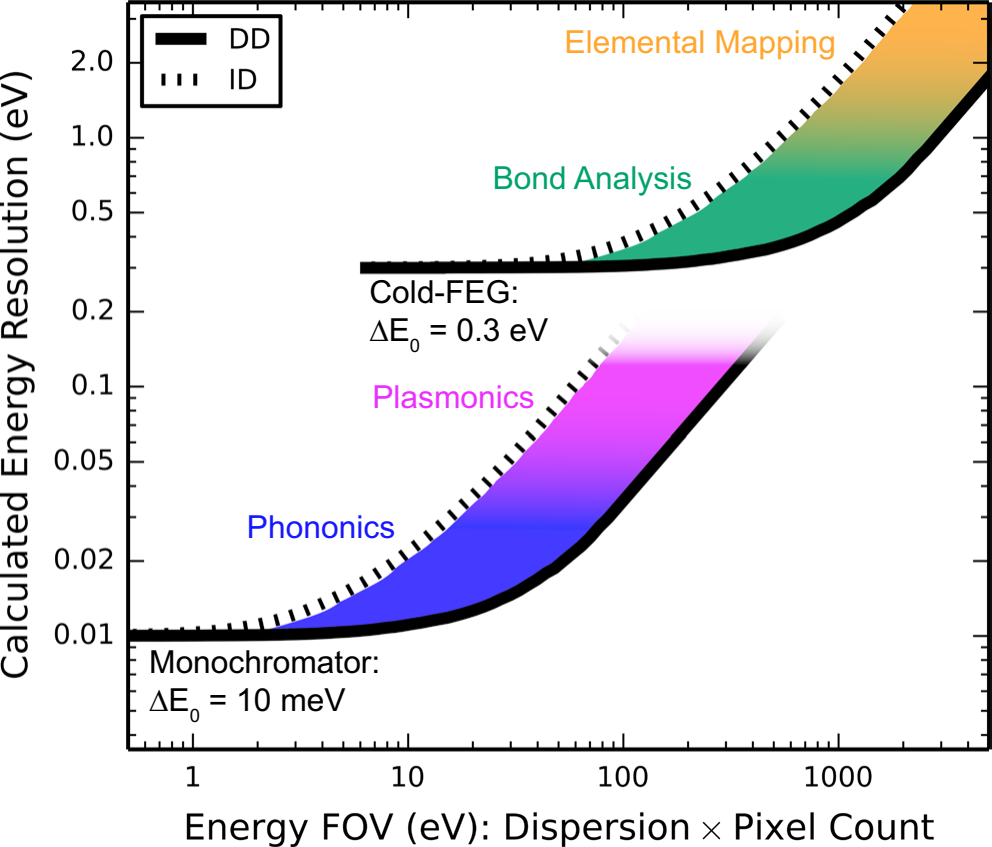
Calculated energy resolution plotted against energy FOV for both sensors for two different electron sources. The required energy resolutions for various EELS applications are identified. The shaded regions represent combined energy resolution/FOV which is accessible with the DD sensor but not the ID sensor.

While the data in Fig. 2 was acquired with a Schottky source with *∆E*_*0*_ = 0.6 eV, the sharp PSF and reduced pixel size of DD offers benefits regardless of the emission source. To illustrate this point, we simulate the performance of DD and ID sensors for a cold-FEG with *∆E*_*0*_ of 0.3 eV and a monochromated source with *∆E*_*0*_ of 10 meV. Figure 3 displays plots of energy resolution against FOV, parameterized by the dispersion (see methods for simulation details). For very small FOV (high dispersion), resolution is limited by the source. As the dispersion is reduced and the FOV increases, the resolution offered by the ID sensor quickly rises owing to signal spreading between channels. Conversely, the resolution offered by the DD sensor remains close to the value of *∆E*_*0*_ for a much broader range of energy FOV. Overlaid on the data are various EELS applications positioned along the *y*-axis according to their required energy resolution^41^. The shaded areas between the ID and DD curves represent values of combined energy resolution and FOV available with DD but not accessible with the ID sensor. Clearly, the DD sensor offers great improvements in combined energy resolution/FOV.

### Signal to Noise Ratio and Detective Quantum Efficiency

SNR and DQE are vital when either electron dose must be limited for radiation sensitive specimens or time-resolution must be minimized for *in situ* characterization; here we show that electron counting significantly improves spectral DQE. First we define SNR as the average number of primary electrons (pe) per energy channel *N* divided by the signal standard deviation after background subtraction σ for a uniformly power-law decaying region of the spectrum. This sets the noise floor that any EELS edge signal must rise above to be detected. Spectral noise has several components: shot noise, gain noise, read-out noise, and Fano noise^42, 43^. Shot noise originates from particle counting statistics and introduces noise of *N* ^*1/2*^. Gain noise, related to slight differences in gain between channels (or errors in the gain correction), scales linearly with *N* and is characterized by a proportionality constant *v*. Read-out noise *n*_*r*_, due to numerous electronic processes, is added to each spectra as it is physically detected and is independent of *N*. Fano noise arises from variations in the signal generated (and collected) per primary electron and is generally taken to be proportional to the shot noise in simple models. This noise is quantified by the Fano factor *F*. For ID sensors, *F* relates to photon generation/collection, and for DD sensors, *F* relates to electron-hole generation/collection and is referred to as Landau noise. By adding these noise contributions in quadrature, the following expression allows calculation of SNR^42, 44, 45^

2 $$SNR=N/\sigma =N/\sqrt{N/{s}^{2}+{(vN)}^{2}+m{n}_{r}^{2}+NF/{s}^{2}}$$

where *m* is the number of summed frames. Because shot noise and Fano noise originate prior to signal detection, the PSF of each sensor will partially smooth these fluctuations, resulting in a reduction of measured noise. A complete description of this effect would require convolution with the detector PSF, but for clarity we use a single parameter *s* to describe the noise reduction^46^. An ideal detector has *s* = 1 while a detector with *s* > 1 has an average mixing of the signal among *s* detector channels.

To evaluate SNR, EEL spectra were recorded across a broad range of *N* for both sensors. The ID sensor was operated at its minimum (1×) and maximum (130×) values of vertical binning. Higher binning values reduce read-out noise and time but increase gain noise. Thus 1× binning is generally favored for high doses while 130× binning is favored for high-speed, low-dose acquisition. The CCD summing well ADC was run at 1 MHz to minimize readout noise.

**Figure 4:**
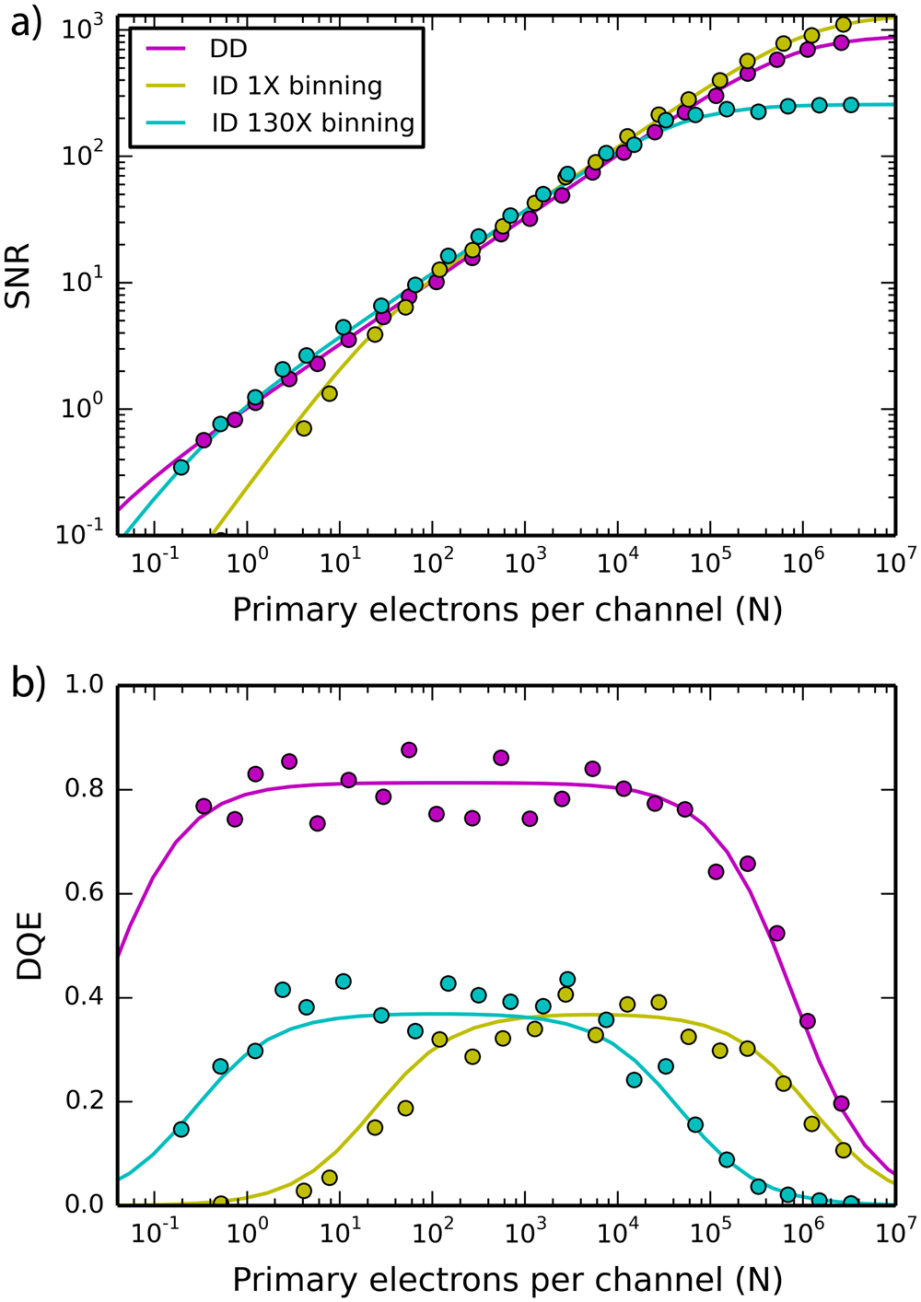
(**a**) SNR for each sensor as a function of total electron counts per energy channel. Circles represent experimental data, and the solid lines represent calculated SNR using equation (2). (**b**) DQE as a function of electron counts per energy channel. Calculations were performed with equation (3).

The circular markers in Fig. 4(a) show experimental SNR as a function of *N* for both the DD and ID sensors, and the solid lines show calculated SNR using equation (2). For the calculated data, the experimentally determined *n*_*r*_ was used. *F* was set to zero for the DD sensor since electron counting eliminates this noise source, and *F* was taken from ref. 47 for the ID sensor. Values for *s* and *v* were then fit to the experimental data. All three SNR curves show the same essential behavior. For moderate doses, shot noise dominates and SNR is approximated by $\sqrt{N{s}^{2}/(1+F)}$. In this regime the ID sensor shows the same SNR for both binning settings since binning doesn’t affect *F* or *s*, and the ID sensor shows higher SNR than the DD sensor due to increased shot noise smoothing (*s* = 1.95 for ID and *s* = 1.03 for DD). For low electron doses, shot noise decreases and the relative weight of read-out noise increases. In the limit of *N* = 0 (read-out noise limited acquisition), SNR will follow $\,N/\sqrt{m}{n}_{r}$. Taking the transition from $SNR\propto \sqrt{N}$ to $SNR\propto N$ as the dose where read-out noise equals shot noise, the transitions occur at *N* = 0.025, 0.19, and 14 pe/channel for the DD sensor, ID sensor at 130× binning, and ID sensor at 1× binning, respectively. Such a transition is observed for the ID sensor experimentally, but given the range of *N* studied here, the DD sensor remains shot noise limited. At high *N*, gain noise dominates and SNR approaches a limiting value of $\,1/v$; a leveling off of SNR is observed with both sensors. From the fitted values of *v*, we report the value of *N* where gain noise equals shot noise: 1.7 × 10^6^ for the ID sensor at 1× binning, 8.5 × 10^5^ for the DD sensor, and 6.9 × 10^4^ for the ID sensor at 130× binning. In this analysis, we have neglected the noise of the reference images used to account for the offset and normalization of each detector pixel, and it has been assumed these references can be measured with vanishingly small noise. However, if this noise is significant, it will add proportionally to the number of frames summed, which for the DD sensor is particularly troublesome due to the large number of frames needed to accumulate a high signal image. The saturation of the DD SNR at large *N* is a direct consequence of the noise in the reference images and can, in principle, be reduced.

While not immediately obvious in the SNR data, the reduction in *n*_*r*_, *F*, and *v* (for the comparison to 130× binning) offered by the DD sensor dramatically increases spectrum quality. To quantify this improvement, we evaluate the DQE for each EELS sensor. The DQE is a critical parameter for determining a detector’s performance, and it is defined as the ratio of the square of the recorded SNR to the square of the input SNR:$\,DQE=SN{R}^{2}/SN{R}_{IN}^{2}$. An ideal detector has a DQE of unity; however, the DQE of real detectors is always smaller.

The DQE of the K2 has previously been compared to various ID sensors by the biological and detector physics communities. Such studies evaluate the DQE in the frequency domain using experimental measurements of detector MTF and noise power spectra^36, 38^. We utilize a heuristic DQE methodology^48^ which is more commonly used for EELS analysis^42, 46^. This approach is less quantitative than the frequency based formalism and reduces the detector MTF to a single-valued parameter which neglects the differing distributions of the various noise sources; however, the method used here offers several advantages: 1) it presents DQE as a function of electron counts which is relevant for EELS, 2) it may be determined directly from EELS data, and 3) the effects of the various noise sources – and their differing magnitude between sensors – are easily identified.

Here SNR_IN_ is defined as the SNR recorded with an ideal detector with the same shot noise smoothing, $SN{R}_{IN}=s\sqrt{N}\,$ ^42^, where the parameter *s* represents the PSF smoothing of the pixelated detector as described above. Using equation (2), the DQE of the ID sensor then becomes

3 $$DQE=N/{\sigma }^{2}{s}^{2}={(1+F+{s}^{2}[m{n}_{r}^{2}/N+{v}^{2}N])}^{-1}$$

For DQE analysis of the DD sensor in counting mode, the effect of counting losses must be also considered. With the K2 counting algorithm, 13% of electrons pass through the sensor without being detected, leading to a quantum efficiency (QE) of 87%^49^. Additionally, two electrons striking the same area of the detector within the same readout period cannot be differentiated, which leads to coincidence loss at elevated dose rates^21, 49^. As such, we define SNR_IN_ for the DD sensor as the expected SNR for a sensor with no counting losses:$\,SN{R}_{IN}=s\sqrt{N/QE(1-CL)}$, where CL is the estimated fraction of coincidence loss given the dose rate^38^. The end result is that the right hand side of equation (3) is multiplied by a factor of 0.82.

Figure 4(b) shows the DQE of each sensor across a broad range of *N*. Circular markers represent measured data, and the solid lines show the calculated data. The DD sensor shows higher DQE for all values of *N*. In particular, the reduced read-out noise of counting mode results in excellent DQE down to *N* = 0.1 for the DD sensor. Owing to Fano noise, the ID sensor does not surpass a DQE of 0.4. Practical consequences of these results are described in detail within the outlook section.

### Spectrum Imaging

Spectrum image acquisition of a Pb(Zr_0.2_Ti_0.8_)O_3_ (PZT)/(La_0.2_Sr_0.8_)MnO_3_ (LSMO)/SrTiO_3_ (STO) heterostructure was conducted with both the DD and ID sensors. EELS is an invaluable characterization tool for such oxide devices where minute changes in local valence and stoichiometry are critical to material properties. Compared to soft matter, oxides are relatively tolerant to electron irradiation, but beam-induced electronic and physical sample degradation may still occur^32, 33, 50–52^. Additionally, spatial resolution is paramount, thus reduced SI acquisition time is important to limit both sample drift and damage^34^. To this end, SIs were acquired with the short dwell time of 5 ms. Additional SI acquisition parameters are displayed in Table 2. Note that to achieve 200 spectra/s with the ID sensor, the duty cycle is reduced to ≈86% owing to a 0.78 ms read time resulting in an exposure time of 4.3 ms for the ID sensor. The ID sensor was operated at 130× binning to maximize SNR and live time of the acquisition given this short dwell time.

**Table 2:** Parameters used for SI acquisition.

Common parameters					
Pixel size	1 Å				
Map area	275 × 75 Å^2^				
Spectral rate	200 spectra/s				
Probe size	<4 Å				
Collection semi-angle	45 mrad				
**For Ti ELNES**					
Probe current	117 pA				
Source res. (*∆E*_*0*_)	0.6 eV				
Convergence semi-angle	16 mrad				
**For Sr Mapping**					
Probe current	134 pA				
Source res. (*∆E*_*0*_)	1.5 eV				
Convergence semi-angle	8 mrad				
**Sensor specific parameters**					
	DD	ID			
Duty cycle	100%	86%			
eV/channel	0.5	0.125	0.25	0.5	1.0
FOV (eV)	2 k	250	500	1 k	2 k
ZLP FWHM (eV)	0.5	0.75	1.0	1.5	3.0

The measured ZLP FWHM values correspond to the Ti ELNES condition where the source has *∆E*_*0*_ = 0.6 eV. The source was measured with the DD sensor at a dispersion of 0.05 eV/channel. The measured ZLP FWHM with the DD sensor at 0.5 eV/channel is less than *∆E*_*0*_ due to the sharp sensor PSF and aliasing. Elemental mapping necessitated increasing the extractor voltage to push the Schottky source’s secondary electron peak^64^ past the Sr L edge. The increase in extractor voltage resulted in a *∆E*_*0*_ of 1.5 eV.

#### Fine Structure Analysis

**Figure 5:**
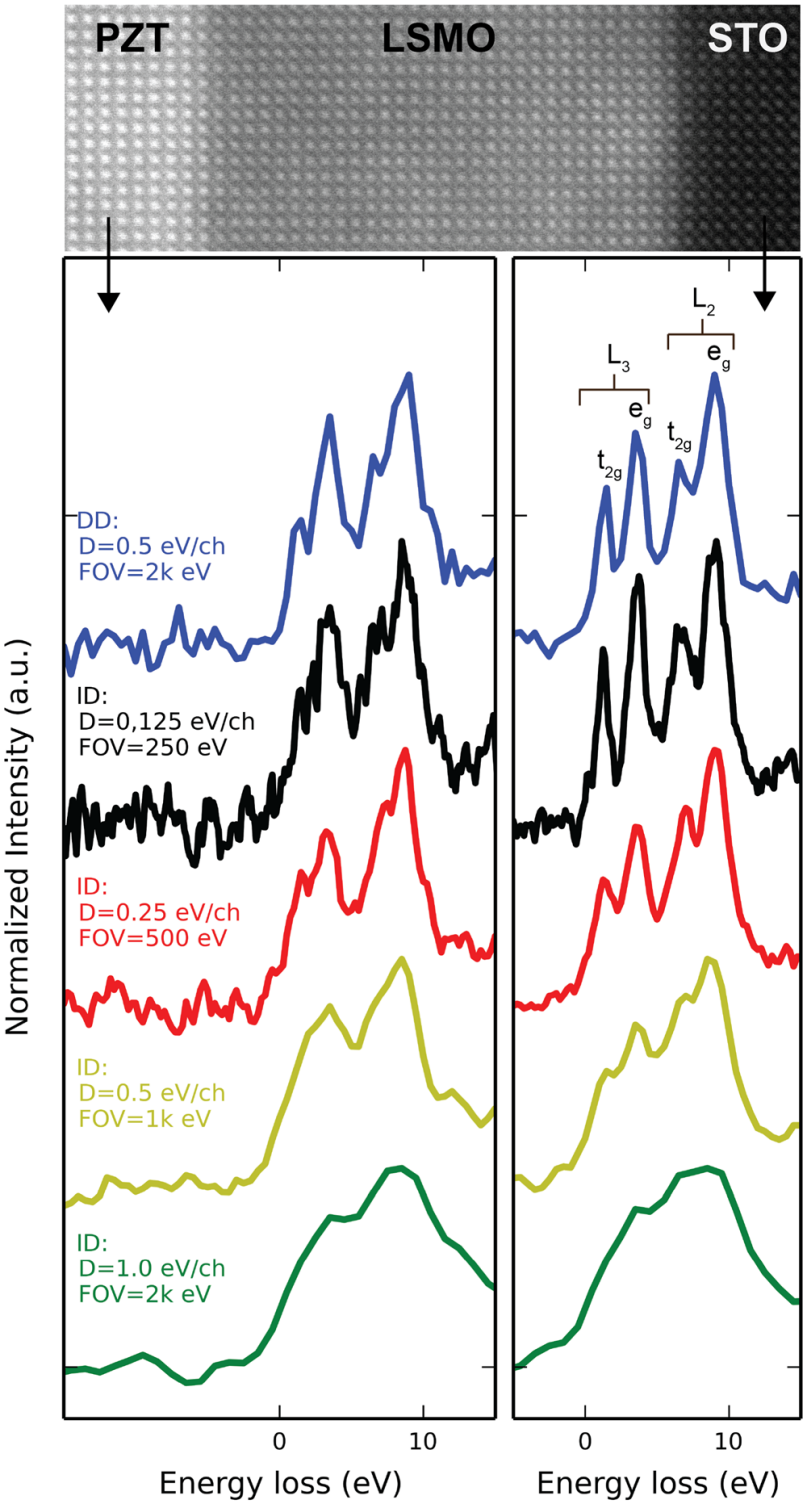
EEL spectra extracted from SIs of a PZT/LSMO/STO heterostructure. A HAADF image of the sample is shown. All spectra were background subtracted and normalized to the integrated L_2,3_ edge intensity. The edge onsets were aligned and this position was set to zero on the energy-loss axis.

From each SI, individual spectra with 8 × 8 Å^2^ areas (giving net exposures of 0.32 and 0.28 seconds for DD and ID, respectively) were summed within the PZT and STO layers to compare ELNES acquisition with each sensor. Figure 5 shows a HAADF image of the heterostructure, as well as summed spectra with the energy range focused on the Ti L edge. In perovskite titanates, the Ti L edge displays a rich fine structure. Spin-orbit coupling of Ti 2*p* orbitals splits the edge into L_2_ and L_3_ transitions, each of which are further split by the octahedral crystal field, allowing transitions into final 3*d* states of *e*_*g*_ and *t*_*2g*_ symmetry. Subtle changes in structural symmetry^53^ and oxidation state^54^ affect the relative weight and energy position of these peaks.

From the SI acquired with the DD sensor, the spectrum extracted from the STO layer clearly shows 4 distinct peaks in the Ti L edge. The spectrum extracted from the PZT shows a distinctly different Ti fine structure, with the relative weight of *t*_*2g*_ features strongly reduced compared to STO. For the ID sensor operated at 1.0 eV/channel dispersion (equivalent FOV to DD sensor at 0.5 eV/channel), splitting of the L_2_ and L_3_ edges is barely resolved, and splitting of *e*_*g*_ and *t*_*2g*_ transitions is entirely absent. The difference in Ti fine structure between layers is not identified. For spectra acquired with the ID sensor at a dispersion of 0.5 eV/channel, *e*_*g*_ and *t*_*2g*_ splitting is not resolved within the PZT layer and hardly resolved for the STO; there is a clear reduction in measured energy resolution compared to the DD sensor at the same dispersion. When the ID sensor is operated at 0.25 or 0.125 eV/channel, the Ti fine structure in the STO is collected with comparable quality to the DD sensor at 0.5 eV/channel. For this equivalence in energy resolution, the ID sensor possesses a greatly reduced energy FOV. Additionally, the increased dispersion spreads the same number of electrons over more pixels lowering *N* and SNR on a per channel basis. This reduction in *N*, combined with the lower duty cycle and lower DQE of the ID sensor, increases spectrum noise. For the ID sensor at a dispersion of 0.125 eV/channel, this increase in noise corrupts the Ti L fine structure in the PZT, where the Ti concentration is reduced relative to the STO. Thus the DD sensor operated at 0.5 eV/channel provides improved fine structure analysis compared to the ID sensor operated at 0.125 eV/channel dispersion, given this high spectral rate and emission source.

#### Elemental Mapping

From each acquired SI, elemental maps were constructed. For the ID sensor, simultaneous detection of Ti and Sr may only be achieved with a dispersion of 1 eV/channel, which provides the necessary 2k eV FOV. As such, we only present elemental maps from the ID sensor at 1 eV/channel which we compare to the DD sensor at 0.5 eV/channel (also 2k eV FOV).

**Figure 6:**
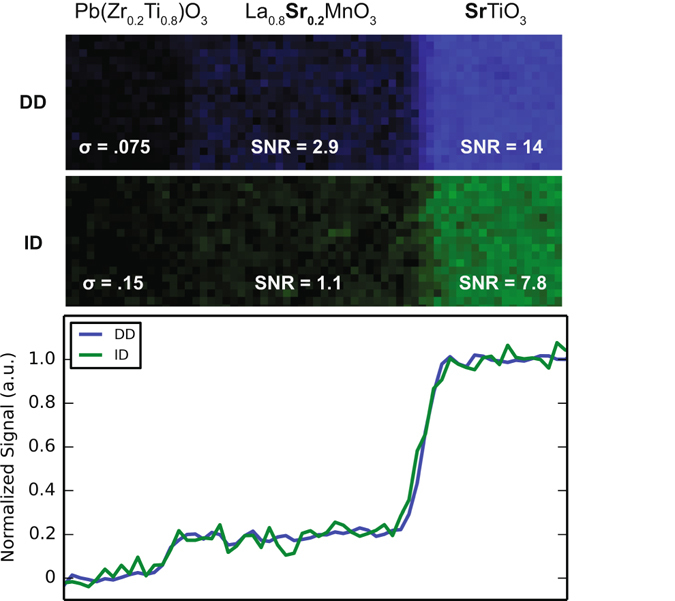
Elemental maps of Sr, acquired with each sensor. For the line profiles in the bottom panel, counts were integrated parallel to the interface and normalized such that the average Sr signal within the STO layer was 1.

Figure 6 shows signal intensity maps for the Sr L edge acquired with each sensor after spatially binning the SI by 4. Equivalent procedures were used to extract the maps (See methods). We calculate the Sr signal SNR (average signal divided by the standard deviation) within the STO and LSMO layers, and the signal standard deviation within the PZT layer after normalizing the signal map by the integrated Sr intensity (Fig. 6). In the STO and LSMO layers, the DD sensor shows a 1.8× and 2.6× increase in SNR, respectively, and in the PZT layer, DD provides a 2.0× noise reduction. These improvements are clearly visible in bottom panel of Fig. 6, where the signal intensity was integrated vertically (parallel to the interface), normalized, and plotted versus position. These results are attributed to the higher DQE offered by the DD sensor and represent an increase in elemental mapping performance with electron counting.

### Outlook

#### Applications

As we have demonstrated, the narrow PSF and reduced pixel size of DD provides an outstanding combination of energy resolution and energy FOV, and electron counting greatly improves DQE for all measured electron doses. As a result, fast SI acquisition with the DD sensor operated at a single dispersion (0.5 eV/channel) provides improved fine structure analysis compared to the ID sensor optimized for high energy resolution (0.125 eV/channel) while simultaneously offering higher quality elemental mapping compared to the ID sensor optimized for energy FOV (1.0 eV/channel). This increased performance will facilitate complete spectroscopic characterization with a considerable reduction in electron dose.

These advances are particularly promising for beam sensitive specimens, such as biological and soft matter samples. Application of EELS in such fields is narrow because the electron dose required for analysis often exceeds the critical dose for samples of interest^30, 31^. While a material’s damage threshold is fixed, the minimum electron dose *D* needed to reliably detect an edge is strongly dependent of the EELS sensor^30, 31^

4 $$D=\frac{{k}^{2}}{{C}^{2}{\epsilon }(DQE)}$$

where *k* is a constant, determined to be ≈5 by Rose^55^, *C* is the edge contrast (signal to background ratio), and *ε* is the efficiency. While the efficiency of an EELS system is dependent on many variables, the ID sensor will lower total efficiency owing to the CCD charge read-out process. The drop in efficiency (duty cycle) is dependent on the spectral rate. For instance, at 400 spectra/s the ID sensor has a duty cycle of 69%.

The difference in DQE between sensors is a function of electron dose (Fig. 4) as well as signal frequency. For chemical mapping using broad pre- and post-edge energy windows, only the zero-frequency DQE is relevant. For ELNES, energy resolution is vital and thus the high-frequency DQE is pertinent. We generalize equation (3) to provide DQE as a function of frequency *f* ^45^:

5 $$DQE(f,N)=\frac{MTF{(f)}^{2}}{NTF{(f)}^{2}\times (1+F)+m{n}_{r}^{2}/N+{v}^{2}N}$$

where the NTF(*f*) is the noise transfer function normalized such that NTF(0) = 1^47^. For the DD sensor in counting mode, the NTF is assumed to be unity which is a good approximation for low dose rates^38, 49^, and for the ID sensor, the NTF is taken as the Fourier transform of the extracted ID sensor LSF (see methods). For both sensors, the MTF was taken from Fig. 2, such that equation (5) gives the DQE(*f*) for the EELS system, not just the sensor.

Table 3 compares the minimum required doses *D*_ID_ and *D*_DD_ as a function of spectral rate and energy frequency, with the edge contrast fixed. The low dose condition of *N* = 10 is assumed. For elemental mapping at 400 spectra/s, the ID sensor requires 3.2× the dose as the DD sensor, and for ELNES at 400 frames/s, the ID sensor requires 5.2× the dose. This calculation does not consider the improved energy FOV offered by DD. If multiple SIs are required with the ID sensor to cover a larger energy range, obviously the dose will increase proportionally. These reductions in minimum electron dose offered by the DD sensor may enable the application of EELS to a range of materials previously deemed too sensitive for spectroscopic analysis.

**Table 3:** Ratio of minimum electron dose for the ID sensor compared to the DD sensor as a function of energy frequency and spectral rate.

Application	Elemental mapping		ELNES	
Energy frequency	*f* = 0 eV^−1^		*f* = 0.25 eV^−1^	
spectral rate (Hz)	100	400	100	400
*D*_ID_/*D*_DD_	2.4	3.2	3.9	5.2

The value of DQE(0.25 eV^−1^) assumes a dispersion of 0.5 eV/channel and source energy width of 0.6 eV. The calculations assume 130× binning with ID sensor.

Beyond the study of beam sensitive materials, DD EELS will have a significant impact for time resolved spectroscopy. Equation (5) is easily rewritten to express the time resolution *∆t* of EELS:

6 $${\rm{\Delta }}t=\frac{{k}^{2}}{J{C}^{2}{\epsilon }(DQE)}$$

where *J* is the current density. Considering just the DQE term, the DD sensor will reduce *∆t* by a factor of 2.2 for elemental mapping and, for ELNES, by a factor of 3.6. Additionally, the readout time of the CCD will add 0.78 ms to *∆t* for the ID sensor. This improvement will aid *in situ* observation of chemical and physical processes too rapid to be tracked dynamically with conventional EELS.

Trace element analysis will also benefit from the improved DQE. The minimum detectable atomic fraction (MAF)^48^ is proportional to ε^−1/2^DQE^−1/2^. For mapping at 400 frames/s, the MAF is reduced by 45% going from the ID to DD sensor. Extended edge fine structure analysis may also benefit from this result.

#### Beam Current and Energy Considerations

A drawback of electron counting is the limited dose rate. For the full frame readout rate of the described DD sensor, coincidence loss for dose rates below 4 e^−^ pixel^−1^ s^−1^ is minimal, resulting in a near-linear relation between incident and counted electrons with a quantum efficiency of 87%^49^. Above 4 e^−^ pixel^−1^ s^−1^, the relation between incident and counted electrons becomes nonlinear. At 10 e^−^ pixel^−1^ s^−1^ the K2 has 11% coincidence loss, and at 32 e^−^ pixel^−1^ s^−1^ the loses grow to 29%^21^. While the preceding data corresponds to the K2 operated at 300 kV, counting losses at 200 kV are similar^36^. The main drawback of coincidence loss is a decrease in the detector’s low frequency amplitude response, although this effect may be recovered (with added signal noise) for current densities up to 32 e^−^ pixel^−1^ s^−1^, since the incident to counted electron relation is quantified^49^. Such a correction would have to be performed on the full image (prior to projection) requiring firmware modifications to the current implementation.

For EELS acquisition of core-loss edges, current densities may practically be kept below 32 e^−^ pixel^−1^ s^−1^ and often below 4 e^−^ pixel^−1^ s^−1^. For the SIs acquired here with 0.5 eV/channel, a sample thickness of 0.5 mean free paths (MFP), an energy range of 380–2235, and a beam current of ≈130 pA, the highest dose occurred at Ti L edge, which gave 7800 e^−^/channel/s. Spreading this dose across the 4k pixels orthogonal to the axis of dispersion (this requires use of the 5 mm spectrometer aperture) gives 1.9 e^−^ pixel^−1^ s^−1^, though in practice the intensity will be concentrated at the center of the detector due to the non-uniform angular distribution of the electrons entering the spectrometer. The beam current could be increased 2× to 260 pA while maintaining negligible counting losses, or increased to 2 nA while staying within the range where nonlinearity could be corrected.

Acquisition of low-loss and valence/vibrational EELS with electron counting can also be managed. Performing low-loss EELS on the oxide heterostructure studied here with a current of 130 pA, a dispersion of 0.05 eV/channel (energy FOV = 100 eV) and the ZLP off the detector, a maximum dose of 56,000 e^−^/channel/s is observed, corresponding to an idealized maximum arrival rate of 14 e^−^ pixel^−1^ s^−1^. For vibrational and valence EELS with monochromated beams, it is advantageous to directly measure the ZLP. For these applications a beam current 1 pA and energy spread of ≈ 10 meV is practical^3, 56^. For such conditions, the ZLP apex will carry a dose of ≈1.5 × 10^5^ electrons/eV. With a dispersion of 0.5 meV, the maximum dose rate will be below 20 e^−^ pixel^−1^ s^−1^ while providing a 2 eV FOV. For a simple ZLP energy reference, counting losses at the tip of the ZLP are acceptable, and the FOV may be increased to 10 eV with a dispersion of 2.5 meV/channel while keeping the dose rate below 100 e^−^ pixel^−1^ s^−1^.

In the event that a very high intensity feature must be recorded such as the ZLP, coincidence losses may be reduced by modulating the duty cycle of the detector via per frame shuttering. The current incarnation provides per frame shuttering down to 1 μs (duty cycle = 0.04%) using a high-speed electrostatic shutter in the GIF. This shuttering must be carefully synchronized with the rolling read of the sensor. This method is effective at avoiding saturation of the signal (coincidence losses); however, this shuttering is after the sample so the dose on the sample is unchanged. Efficient gun shuttering would allow the user to optimize the electron arrival rate at the DD sensor without unnecessary sample radiation.

Lastly we discuss the role of beam energy. The MAPS design is best suited for high beam energies^57^, and the advantages in EELS performance over conventional ID sensors reported here will likely decrease at lower accelerating voltages. Conversely, hybrid pixel sensors – an alternative DD design – are optimized for low beam energies^58, 59^. To our knowledge, hybrid pixel sensors have not been applied to EELS; however, this DD technology could be advantageous for low-dose chemical analysis for specimens which suffer from knock-on damage.

## Conclusions

We have evaluated the performance of electron counting with DD for EELS by comparing the Gatan K2 Summit with the Gatan US1000FTXP. Our findings show that the improved PSF and pixel density of the DD sensor provides a remarkable combination of energy resolution and energy FOV, which will facilitate simultaneous elemental mapping and fine structure analysis. Counting mode spectroscopy, enabled by the fast electronics and pixel design of the DD sensor, largely eliminates read-out and Fano/Landau noise, allowing acquisition of shot noise limited spectra down to extremely low doses. These benefits promise to enable or enhance a broad array of applications including trace element detection and analysis, low-dose chemical mapping, time resolved EELS, and analysis of high energy-loss events.

## Methods

### Experimental Setup

To compare the DD and ID sensors, both the K2 and US1000FTXP were mounted on the same spectrometer. The US1000FTXP was mounted at the back of a Quantum Gatan Imaging Filter (GIF)^12^ in the standard position and is equipped with a U-Type scintillator (12.6 counts/primary electron at 200 kV). The K2 was mounted on a retractable stage allowing it to be inserted in front of the US1000FTXP. The electron optical alignment of the GIF is individually optimized for each detector, and the saved alignments are automatically loaded when the active detector is changed. For the highest energy resolution, it is often necessary to fine tune the spectrometer focus after changing the setup of the GIF. Automation of the focus process is provided, but many operators do a final manual touchup of the fine focus. It is recommended the focusing is done with the highest practical attenuation factor to limit exposure of the sensor for both DD and ID configurations. The GIF was installed on a JEOL 2100 F TEM, equipped with Schottky source and a high-resolution pole piece with C_S_ = 1.0 mm.

### K2 Firmware Modifications

To enable rapid, real-time viewing of spectra captured with the K2, extensive firmware modifications were required. The K2 has an electronic read-out rate of 50 MHz and constantly acquires frames at 400 Hz (2.5 ms/frame), each frame being ~16 megapixels (3838 × 3710). The K2 Summit possesses dedicated hardware to process this large data load. To reduce data sent to the host computer to a manageable rate in full frame imaging mode, the K2 hardware, after discriminating and electron counting, sums 40 frames which the host computer receives at 10 Hz. For highest spectroscopy performance, each 2.5 ms frame needs to be sent to the host computer which is not practical with existing hardware. To address this problem, the K2 summit processor firmware was adapted such that each frame is first discriminated and counted and then projected to a 4k × 1 spectra within the K2 processor, with the projected data then sent to the host computer at 400 Hz. This adjustment drops the data rate to the host computer by a factor of approximately 500, increasing the spectral rate to the computer from 10 to 400 Hz. This firmware modification was necessary for practical spectrum imaging with the K2 where it is imperative that every spectrum is recorded.

To address over exposure of the K2 under high beam current conditions, the output of the camera shutter was synchronized to the rolling readout of the sensor which allows the intensity of the spectrum to be attenuated before reaching the sensor. Provided the spectrum is attenuated sufficiently to keep the camera in the counting regime with low coincidence loss, the ZLP and low-loss can be recorded without damaging the sensor.

### General EELS Acquisition

For all acquisitions, the TEM accelerating voltage was 200 keV. For ZLP acquisition, the GIF entrance aperture was 2.5 mm, and for SNR analysis and SI acquisition, the GIF entrance aperture was 5.0 mm. We note that to take full advantage of the 4k pixels orthogonal to the axis of dispersion, the 5.0 mm aperture must be used. For ZLP analysis, the beam current was 30 pA, for ELNES the beam current was 117 pA, and for elemental mapping the beam current was 134 pA. The beam current was determined by imaging the unscattered STEM bright field disk with the K2 in counting mode while operating the GIF in imaging mode and assuming a quantum efficiency of 87% and no counting losses. The collection semi-angle for EELS was set to 45 mrad. For ELNES, the convergence semi-angle was 16 mrad, and for elemental mapping it was 8 mrad.

For SIs and spectra acquired with the US1000XPFT for SNR analysis, spectra were gain corrected and the detector ADC clock was set to high quality, 1 MHz mode. For SI acquisition, 130× binning was used. For ZLP analysis, the clock was set to high speed (10 MHz mode) and 1× binning was used to avoid saturation. A gain reference was acquired directly prior to the experiments. The gain reference was performed with a target intensity of 21,000 and 40 averaged frames (http://www.eels.info/resource/protocol/prepare-gain-reference). A high quality dark correction was applied to all spectra (see Gatan Microscopy Suite help file). For the 275 × 75 pixel SIs, the high quality dark correction resulted in the averaging of 428 frames.

### ZLP Acquisition

For ZLP acquisition with the DD sensor, the GIF electrostatic shutter was set to only allow 0.041% (1 μs live time) of the electrons through to the K2. This ensured that the ZLP did not exceed 4 e^−^ pixel^−1^ s^−1^, which would have affected the ZLP measurement. For the US1000FTXP, the exposure was adjusted based on the dispersion. This varied from 10^−3^ s for 0.125 eV/channel to 10^−4^ s for 1 eV/channel. In each instance, the CCD was at ≈1% or less saturation. For each sensor and dispersion, many ZLPs were collected, aligned, and summed to avoid broadening from high tension drift.

### Extraction of MTF

We modelled the system MTF (the system includes contributions from the source and sensor) by fitting the acquired ZLPs to a sum of 3 Gaussians

7 $$ZLP(eV)=\sum _{\lambda }{C}_{\lambda }\exp (e{V}^{2}/{\lambda }^{2})/\pi \lambda $$

where λ is a fitted length parameter, and C_λ_ is a fitted coefficient. With these fitted values, the MTF was analytically extracted with the following expression

8 $$MTF(\omega )=\sum _{\lambda }{C}_{\lambda }\exp (-{\pi }^{2}{\lambda }^{2}{\omega }^{2}/4)$$

where *ω* is in terms of fraction of the Nyquist limit^60^.

### Extraction of LSF

Sensor line spread functions (LSF) were used to produce the data in Fig. 3 and to extract the noise transfer function for the ID sensor. To extract each sensor’s LSF, first the electron source energy spread was measured using the DD sensor at a dispersion of 0.05 eV/channel. A sum of three Gaussians was fit to the data to obtain an analytic representation of the electron source energy spread. This analytic function was then binned (discretized or downsampled) according to the experimental dispersions of 0.125, 0.25, 0.5, and 1.0 eV/channel. At a given dispersion, convolution of the binned electron source energy spread by the sensor LSF should produce the experimentally measured ZLP. The sensor LSFs were thus determined by a least-squares fit, with experimental ZLPs from all dispersions equally weighted in the least-squares fitting procedure.

### Resolution and FOV simulations

The data in Fig. 3 was generated as follows: 1) extract each sensor’s LSF from the data shown in Fig. 2; 2) generate simulated ZLPs with equation (1) using a single Gaussian as in the input electron source energy spread and a broad range of working dispersions; 3) fit a sum of Gaussians to the simulated ZLPs and take the FWHM of the fitted function as the energy resolution; 4) determine the FOV by multiplying the dispersion by the pixel count of each sensor.

### SNR Analysis

Spectra were acquired from a STO sample with both sensors as a function of *N*. A 100 eV energy-window ranging from 1300 to 1400 eV was selected for SNR analysis since this energy range was well removed from any core-loss edges, allowing accurate background subtraction. Both sensors were operated with a dispersion of 0.5 eV/channel. For SNR analysis, EELS was acquired in STEM mode with a beam current of several nA; the exact value was not measured.

Sensor readout noise was determined by subtracting two spectra acquired with the beam blanked, taking the standard deviation, and then dividing by $\sqrt{2}$. This process was repeated 3 times, and the averaged values were found to be 0.163, 0.437, and 3.57 pe for the DD sensor, ID sensor at 130 × binning, and ID sensor at 1 × binning, respectively.

Since the DD sensor constantly acquires frames at 400 Hz, the value of *m* (number of frames summed) in equations (2) and (3) was set by the exposure time. For the ID sensor, a single exposure (*m* = 1) was used until the detector began to saturate. For the ID sensor at 130 × binning, frame summing was necessary past *N* ≥ 10^3^, and at 1 × binning frame summing was necessary past *N* = 10^5^.

### HAADF Image Acquisition

HAADF images were acquired by summing and averaging 10 cross correlated frames with 5 μs dwell time. This was achieved using the StackBuilder plugin by Bernhard Schaffer^61^.

### SI Analysis

For the fine structure analysis shown in Fig. 5, spectra were background subtracted using a power-law fit from 420–450 eV. For elemental mapping of Sr, a power-law background fit was used, and the fit range extended from 1750 to 2075 eV. For Sr mapping with the DD sensor, the data was recorded at 0.5 eV/channel, and then binned to 1 eV/channel prior to signal quantification.

### Sample Preparation

The oxide heterostructure^62, 63^ was prepared for TEM *via* a conventional *in situ* liftout process in a dual-beam focused ion beam (FIB) (FEI DB235). Final thinning in the FIB was performed with 5 keV Ga ions, and a post-FIB cleanup was performed with 3 keV Ar ions. The sample had a surface normal corresponding to the perovskite pseudocubic [100] axis. The sample was measured to be ≈0.5 MFPs thick.

### Data Availability

The datasets generated during and/or analyzed during the current study are available from the corresponding author on reasonable request.
